# A simple work flow for biologically inspired model reduction - application to early JAK-STAT signaling

**DOI:** 10.1186/1752-0509-5-30

**Published:** 2011-02-21

**Authors:** Tom Quaiser, Anna Dittrich, Fred Schaper, Martin Mönnigmann

**Affiliations:** 1 Automatic Control and Systems Theory, Ruhr University Bochum, D-44801 Bochum, Germany; 2 Process Systems Engineering, RWTH Aachen University, D-52064 Aachen, Germany; 3 Department of Biochemistry and Molecular Biology, Medical School RWTH Aachen University, D-52074 Aachen, Germany; 4 Systems Biology, Magdeburg Centre for Systems Biology (MaCS), Otto von Guericke University, D-39120 Magdeburg, Germany

## Abstract

**Background:**

Modeling of biological pathways is a key issue in systems biology. When constructing a model, it is tempting to incorporate all known interactions of pathway species, which results in models with a large number of unknown parameters. Fortunately, unknown parameters need not necessarily be measured directly, but some parameter values can be estimated indirectly by fitting the model to experimental data. However, parameter fitting, or, more precisely, maximum likelihood parameter estimation, only provides valid results, if the complexity of the model is in balance with the amount and quality of the experimental data. If this is the case the model is said to be identifiable for the given data. If a model turns out to be unidentifiable, two steps can be taken. Either additional experiments need to be conducted, or the model has to be simplified.

**Results:**

We propose a systematic procedure for model simplification, which consists of the following steps: estimate the parameters of the model, create an identifiability ranking for the estimated parameters, and simplify the model based on the identifiability analysis results. These steps need to be applied iteratively until the resulting model is identifiable, or equivalently, until parameter variances are small. We choose parameter variances as stopping criterion, since they are concise and easy to interpret. For both, the parameter estimation and the calculation of parameter variances, multi-start parameter estimations are run on a parallel cluster. In contrast to related work in systems biology, we do not suggest simplifying a model by fixing some of its parameters, but change the structure of the model.

**Conclusions:**

We apply the proposed approach to a model of early signaling events in the JAK-STAT pathway. The resulting model is not only identifiable with small parameter variances, but also shows the best trade-off between goodness of fit and model complexity.

## Background

Mathematical models and simulations are central to systems biology. Any mathematical model is only as reliable as the numerical values assigned to its biological parameters, however. Since biological parameters, such as kinetic constants, can often not be measured directly, they must be determined indirectly with parameter estimation (PE) methods. Naturally, results obtained with PE are the more precise, the greater the information content of the experimental data.

It is well-known that there may exist parameters in a model that cannot be estimated by PE at all, i.e. not with a finite error (see e.g. [1-5]). We can asses whether the unknown model parameters of a model may be determined by PE by testing the identifiability of the model. A number of useful definitions of identifiability exist (see [6] for a review). Two concepts are important in the context of the present paper: structural identifiability and at-a-point identifiability. Unfortunately, methods for structural identifiability testing ([5,7-16]) are not widely used for large nonlinear models due to either the computational complexity or the lack of mature computer implementations [17].

Methods for at-a-point identifiability testing [1,3,4,18-22], in contrast, are easier to implement and more widely applicable. Due to their size and nonlinearity, the models treated in the present paper are analyzed with methods for at-a-point identifiability. In the remainder of this article the term identifiability therefore refers to at-a-point identifiability (see Methods section for a definition). We use the eigenvalue method for identifiability testing, because it proved to be computationally efficient and precise in a recent comparison to other methods [2].

If a mathematical model turns out not to be identifiable for the accessible experimental data, we may attempt to replace it by a simplified one. Many methods for model reduction of nonlinear models exist. These include but are not limited to techniques based on detecting and decomposing different time-scales [23-25], sensitivity analysis [21,26,27] and balanced truncation [28-30]. In this contribution our aim is to find an identifiable model. Therefore we propose an identifiability based approach for model reduction. Our iterative approach is not fully automated, but depends on a biologically skilled modeler. Even though the modeler is guided by the proposed approach, he remains in control of the simplification throughout the entire procedure. The work flow proceeds by the following steps: 1) parameter estimation, 2) ranking estimated parameters according to their identifiability, 3) calculating parameter variances, and 4) simplifying those parts of the model that contain the least identifiable parameters. These steps are iterated until an identifiable model results. In the presented case study typical simplifications in step 4) amount to lumping and neglecting reactions that do not affect any model output. We stress that we do not enforce identifiability by merely fixing unidentifiable parameters to values that result in good fits. While parameter fixing helps investigating which parameters could be estimated within the set of unknown parameters [3,31-34], the resulting parameter values must be considered with great care. After all, the values of the fixed unidentifiable parameters remain to be unknowns in these models. In fact, the unidentifiability of these parameters implies infinite error bars have to be assigned to them. Parameter estimation with random number based optimization algorithms can be particularly misleading in this context. These algorithms often suggest there exists an optimization result, even if the PE did not converge to a solution that fulfills optimality criteria. The convergence properties of gradient based optimization methods are, in contrast, closely linked to identifiability. Briefly speaking, convergence of gradient based methods is usually tested by checking first order optimality conditions, which are a necessary condition for local optimality of the least squares parameter estimation to Gaussian approximation and, in turn, a necessary condition for local identifiability of the model (see [2,35] and *Methods*).

The proposed model simplification work flow is applied to a model of Janus kinase (JAK) and signal transducer and activator of transcription (STAT) signaling, which is based on the model by Yamada et al. [36]. The JAK-STAT pathway is of biological importance because it is involved in several key cellular processes such as inflammation, cellular proliferation, differentiation and apoptosis.

We do not use the full JAK-STAT model proposed by Yamada *et al. *[36], since even when all species can be measured noise-free and at high frequency, the full model is by far too detailed [2]. This problem would be even more pronounced if realistic experimental conditions were assumed. Instead of the original JAK-STAT model we use a truncated model that focuses on the early signaling phase (first 15 minutes) of the JAK-STAT signaling pathway before transcriptional feedback occurs.

We apply the proposed iterative work flow to the JAK-STAT model until eventually an identifiable model results. After each iteration step the unknown parameters of the resulting new model are determined by maximum likelihood estimation using simulated data. Consequently, the work flow is applicable *in silico*, i.e. the proposed identifiability analysis may be carried out based on experimental data, but it may also be applied prior to any laboratory measurements. If no experimental data are available, it is important to still incorporate realistic assumptions on which biological quantities could in principle be measured, as well as on which measurement error must be expected. These assumptions on the availability and precision of data are crucial, since they heavily influence identifiability properties.

The proposed work flow extends our earlier work [2], where we assumed that all state variables of the model can be measured to arbitrary precision. For the purpose of the present paper it is important to incorporate stricter assumptions on measurability, sampling rates, and error bars. Moreover, the identifiability criterion used in [2] arguably is difficult to interpret. Here we use a variance based criterion that is both concise and easy to interpret. Finally, the work flow proposed here improves upon the identifiability method used in [2] in that it is iterative and uses multi-start parameter estimation to mitigate the problem of convergence to local minima in non convex least squares optimization.

## Methods

The first part of this section focuses on the description of the JAK-STAT model. After introducing the general system class, the particular JAK-STAT model used throughout the paper is discussed. The second part of the section introduces the proposed model simplification workflow and its building blocks.

### Modeling

#### System class

We consider mathematical models *M *of the form

(1) $$\begin{array}{l} \dot{x}(t)=f(x(t),p,u(t)),\;x(0)=x_{0}\\ y(t)=h(x(t),p,u(t)), \end{array}$$

where $x\in {\mathbb{R}}^{n_{x}}$, $p\in {\mathbb{R}}^{n_{p}}$, $u\in {\mathbb{R}}^{n_{u}}$ and $y\in {\mathbb{R}}^{n_{y}}$ denote state variables, parameters, inputs and outputs, respectively. The functions *f *and *h *are assumed to be smooth. One component *p_i _*of the parameter vector *p *corresponds to one biological parameter such as a kinetic constant. In our example the initial conditions are known. Note that this might not always be the case. The proposed approach can easily be extended to systems with estimated initial conditions (see Additional file 1 supplementary text 1).

We stress that it is important to distinguish state variables *x *from output variables *y*. In a simulation, all state variables *x *can be determined to practically arbitrary precision and recorded at arbitrary sampling rates. In a laboratory experiment, in contrast, many state variables can usually not be measured at all, or not within any relevant precision. Moreover, some state variables can often not be measured separately from others, but only as a sum or, more generally, linear or nonlinear combination. The output variables *y *are introduced to distinguish those quantities that could in principle be measured in a laboratory from the state variables. In terms of the output values *y*, a laboratory or computer experiment results in values at successive points $\text{0}=t_{\text{0}}<t_{\text{1}}<\cdot \cdot \cdot <t_{n_{t}}$ in time

(2) $$y(t_{i}),\;\tilde{y}(t_{i})\;\;i=1,...,n_{t},$$

where *y*(*t*) and $\tilde{y}(t)$ denote simulated output data and experimental data, respectively. Up to measurement noise the outcomes of both a real and a simulated experiment are uniquely determined by the initial conditions *x*(0) = *x*_0 _and the values of the inputs *u*(*t*) from *t *= 0 to the final time $t=t_{n_{t}}$. Input functions *u*(*t*), $t\in [0,t_{n_{t}}]$ also have to be chosen with care, since computer simulations can be run for a much larger class of functions *u*(*t*) than can be realized experimentally. Both the choice of *u*(*t*) and *y *are discussed in detail below.

We use a variable order, variable step size, backward differentiation formula based numerical integrator (DDASPK [37]) to obtain the solution of equation (1). The derivatives of the state variables with respect to the parameters are calculated by adding so called sensitivity equations to equation (1) and integrating the extended equations system (e.g. see [38]).

**Figure 1:**
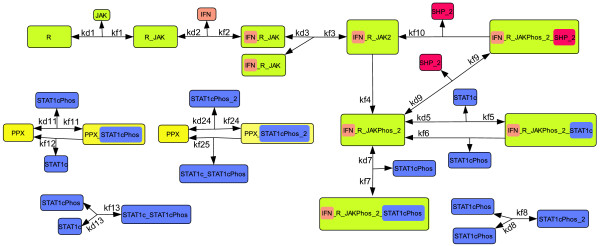
**Truncated JAK-STAT model**. Protein species are depicted as colored rectangles. Arrows between the protein species describe association or dissociation reactions, which follow mass action kinetics. The names of kinetic parameters are written next to the corresponding reaction arrow. A mathematical representation of the model is given in Additional file 1 table S2a and S2b.

We focus on the events that occur in the JAK-STAT pathway during the first 15 minutes after receptor activation. This assumption results in a considerable simplification, since transcriptional feedback and protein synthesis need not be modeled. The model of the JAK-STAT pathway published by Yamada et al. [36] serves as a reference in the present work. Figure 1 sketches those parts of the model published by Yamada et al. [36] that are needed to describe the first 15 minutes after receptor activation. Its signaling steps can briefly be described as follows. In the first step, the receptor (R) associates with JAK to form the R_JAK complex. Binding of interferon-*γ *(IFN) to R_JAK creates the receptor complex IFN_R_JAK, which is able to dimerize. JAK may phosphorylate the dimerized receptor complex, and, as a consequence, the active receptor complex (IFN_R_JAKPhos_2) is formed. Either SH2 domain-containing tyrosine phosphatase 2 (SHP2) or cytoplasmic STAT1 (STAT1c) can bind to this complex. In the first case, the receptor complex is deactivated. In the latter case, the activated receptor phosphorylates STAT1c, a prerequisite to STAT1c dimerization. The remaining parts of the model describe how STAT1c can be dephosphorylated by the cytoplasmic phosphatase PPX, and how STAT1c monomers and dimers can be converted into each other.

#### Choice of input function u(t) and outputs y_i_

**Figure 2:**
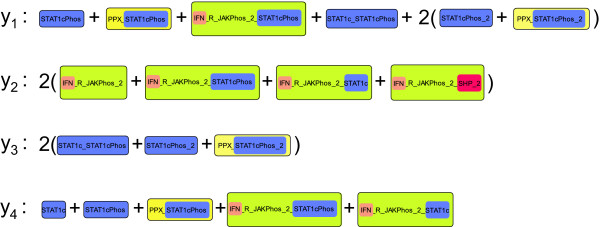
**Model outputs**.

As pointed out above, input functions *u*(*t*), $t\in [0,t_{n_{t}}]$ and output variables *y_i _*have to be chosen with care, whenever simulations are supposed to mimic conditions of realistic laboratory experiments. We choose an input function *u*(*t*) that represents a pulse stimulation with IFN,

(3) $$u(t)=IFN(t)=\left\{ \begin{array}{l} 1,\text{if}\;t\leq 7\min\\ 0,\text{else}, \end{array}\right.$$

where IFN is assumed to be removed completely from the medium in a washing step at time *t *= 7 min. Throughout the paper we consider the outputs *y*_1_,...,*y*_4 _illustrated in Figure 2. Since it is reasonable to assume that these outputs could be measured every minute in a laboratory experiment, we record their simulated values at times *t *= 0 min,..., 15 min and discard all other simulated values. The outputs *y*_1_,...,*y*_4 _correspond to the following quantities: the sum of the concentrations of all phosphorylated STAT1 molecules regardless of their binding or dimerization status (*y*_1_); the concentration of all activated JAK molecules regardless of their binding status (*y*_2_); the concentration of all STAT1 dimers regardless of their binding and phosphorylation status (*y*_3_); and the concentration of all STAT1 monomers regardless of their binding or phosphorylation status (*y*_4_).

By choosing the output *y*_1 _as defined in Figure 2 we implicitly assume that phosphorylated STAT1 molecules can experimentally be distinguished from unphosphorylated ones. Similarly, measuring *y*_2 _requires distinguishing phosphorylated JAK molecules from unphosphorylated ones. We assume the necessary measurements can be accomplished by western blotting with phospho-specific antibodies. Furthermore, output *y*_3 _requires distinguishing dimeric from monomeric STAT1 species. This can be accomplished, if we assume that native SDS-gels, which separate dimers from monomers, are used for Western blot measurements. Finally, we assume that relative phosphorylated JAK and phosphorylated STAT1 concentrations from Western blot measurements can be converted into absolute concentrations by immunoprecipitating the phosphorylated protein with phospho-specific antibodies and parallel Western blotting of a calibrator protein (recombinant JAK or STAT1) with known concentration.

Since Western blot experiments are known to have large standard deviations, we choose a standard deviation of 20% [31]. The choice of experimental noise is critical for the results of any analysis of the parameter variance, since larger measurement noise leads to larger parameter variance.

We stress again that all values *y_i_*(*t_j_*) are simulated values throughout the paper. As pointed out above, however, it is crucial to choose outputs, inputs, and the sampling time which mimic real experimental conditions.

### Model simplification work flow

The model simplification work flow combines delocalized parameter estimation, identifiability testing, variance analysis, and goodness of fit testing. We describe these building blocks first. Note that the work flow can either be started with simulated data from a reference model or with real data.

#### Parameter estimation

Assuming normally distributed and independent observational noise, the solution of the optimization problem

(4) $$\hat{p}=\underset{p}{\arg\;\min}\chi^{2}(p),\text{where}$$

(5) $$\chi^{2}(p)=\sum_{i=1}^{n_{y}}\sum_{j=1}^{n_{t}}{\left(\frac{{\tilde{y}}_{i}(t_{j})-y_{i}(t_{j},p,x_{0})}{\sigma_{ij}}\right)}^{2},$$

results in the maximum likelihood estimate (MLE) of *p*, where *σ_ij _*denotes the standard deviation of data point ${\tilde{y}}_{i}(t_{j})$. The MLE is calculated numerically with the gradient-based sequential quadratic programming software NPSOL5.0 [39]. Since gradient-based solvers for nonlinear problems are attracted by local optima, results strongly depend on starting values. We solve the optimization problem for many starting values on a parallel computing cluster to mitigate this well-known problem. Starting values are sampled with Latin Hypercube Sampling (LHS) [40] (see Additional file 1 supplementary text 2) for an efficient coverage of the sampled space. We will refer to this delocalized parameter estimation approach as *multi-start estimation *for short.

#### Identifiability

The parameter *p_k _*of the model (1) is called globally (locally) structurally identifiable, if for any admissible input *u*(*t*), *p' *and *p" *(*p" *from some neighborhood of *p*'), the equality *y*(*p'*, *u*) = *y*(*p"*, *u*) implies $p_{k}^{'}=p_{k}^{"}$. Global (local) at-a-point identifiability is defined analogously for a fixed reference parameter *p'*. In the remainder of the article the term identifiability refers to at-a-point local identifiability.

It can be shown that identifiability of a model implies uniqueness of the minimizer $\hat{p}$ of (4) [4,35]. Conversely, a model is not identifiable if $\hat{p}$ minimizes (4) and there exists a $\tilde{p}\neq \hat{p}$ such that $\chi^{2}(\tilde{p})=\chi^{2}(\hat{p})$. We can locally test if such a $\hat{p}$ exists by analyzing the neighborhood of a minimum $\hat{p}$ of (4) using the quadratic approximation of χ^2^

$$\chi^{2}(\hat{p})\approx \frac{1}{2}\Delta p^{T}H\Delta p,$$

where $\Delta p=\tilde{p}-\hat{p}$ and *H *is the Hessian matrix of (5). In Gaussian approximation the components of *H *read

(6) $$\begin{array}{c} H_{kl}=\sum_{i=1}^{n_{y}}\sum_{j=1}^{n_{t}}\frac{\partial y_{i}(t_{j},p,x_{0})}{\partial p_{k}}\frac{1}{\sigma_{ij}^{2}}\frac{\partial y_{i}(t_{j},p,x_{0})}{\partial p_{l}}\\ ={\left(S^{T}WS\right)}_{kl}, \end{array}$$

where *W *is the inverse of the measurement variance matrix. For any fixed $\epsilon_{\chi^{2}}$ the inequality $\Delta p^{T}H\Delta p\leq \epsilon_{\chi^{2}}$ describes an ellipsoid that approximates the ϵ-uncertainty region around $\hat{p}$. The axes of this ellipsoid coincide with the eigenvectors *u_i _*of *H*. The length of the *i*th axis is proportional to $1/\sqrt{\lambda_{i}}$, where *λ_i _*denotes the eigenvalue of eigenvector *u_i_*. Since *H *is positive semidefinite in Gaussian approximation, its eigenvalues are nonnegative. If any eigenvalue is close to zero, *λ_i _*≈ 0, the ellipsoid that represents the ϵ-uncertainty region is very elongated in the direction of the corresponding eigenvector *u_i_*. This implies that parameters far apart have almost the same *χ*^2 ^value, or equivalently, at least one parameter has a large variance. Correspondingly, the existence of one or more *λ_i _*= 0 implies at least one parameter has an infinite variance.

We use an eigenvalue-based approach [2,32] to rank model parameters *p_i _*according to their identifiability. This method identifies the smallest eigenvalue *λ*_min _of *H*, searches for the largest component in the corresponding eigenvector *u*_min_, and fixes the corresponding parameter to its estimated value ${\hat{p}}_{i}$. The Hessian is then recomputed with respect to all parameters that have not been fixed, and the procedure is repeated until all parameters are fixed. The order in which parameters are fixed by this approach corresponds to the identifiability ranking of parameters, with the first parameter in the ranking being the least identifiable and the last parameter being the most identifiable one [2,32]. We stress that, in contrast to [2], we do not use the eigenvalue method to distinguish the identifiable parameters from the unidentifiable ones, but only to rank all parameters according to their identifiability. The resulting ranking is then used in the variance analysis as described in the next section. In particular we do not need a cutoff value for small eigenvalues as introduced in [2].

#### Variance analysis

Intuitively, a model is locally identifiable at $\hat{p}$ if the variances *σ*^2^(*p_i_*) of all parameters are small. A variance based identifiability criterion, which is stated more formally below, is preferred over the eigenvalue criterion [2,32] for its simplicity and intuitive interpretation. Variances *σ*^2^(*p_i_*) are determined by running parameter estimations (4) for a large number *N *of starting points in the neighborhood of a minimizer $\hat{p}$ of (4), accepting those estimation results with small *P*-value (defined below), and calculating the mean and variances for the resulting set of accepted parameter estimates. More precisely, we consider an estimation result to be acceptable if it has a significantly small *P*(*χ*^2^|*v*) value [41] (≤ 0.1%, see *Results and Discussion*), where *P*(*χ*^2^|*v*) is the probability for the value of the *χ*^2^-distribution with *v *= *n_y_n_t _*- *n_p _*degrees of freedom to be less than an observed *χ*^2^-value. The variance *σ*^2^(*p_i_*) and the mean ${\bar{p}}_{i}$ are calculated from the accepted estimates according to the following equations.

(7) $$\sigma^{2}(p_{i})=\frac{1}{n_{\text{accept}}-1}\sum_{j=1}^{n_{\text{accept}}}{\left(Q_{ij}-{\bar{p}}_{i}\right)}^{2},$$

(8) $${\bar{p}}_{i}=\frac{1}{n_{\text{accept}}}\sum_{j=1}^{n_{\text{accept}}}Q_{ij},$$

where *n*_accept _is the number of accepted estimates, and *Q *is an *n_p _*× *n*_accept_-matrix that contains these estimates in its columns. Since both variance and standard deviation depend on the mean, we use the scale invariant coefficients of variation $v(p_{i})=\sigma \text{(}p_{i}\text{)}/{\bar{p}}_{i}$[42] to compare variances of different parameters. A *v*(*p_i_*) value of 1, for example, implies the estimates for parameter *p_i _*exhibit 100% standard deviation relative to the mean.

We consider a model to be identifiable, if the coefficients of variation *v*(*p_i_*), *i *= 1,...,*n_p_*, of all parameters are smaller than a bound $\bar{v}$ (smaller than $\bar{v}=0.01$ deviation, see *Results and Discussion*). A value of $\bar{v}=0.01$ implies the parameter estimation step resulted in a relative standard deviation of 1% or less for all parameters. Note that this interpretation of $\bar{v}$ is only valid if the parameter estimation runs cover a sufficiently large neighborhood of the candidate value $\hat{p}$. If the sampled neighborhood is too small, the estimates converge to parameter values that cover the entire sampled neighborhood, and the neighborhood needs to be enlarged. In this case, *v*(*p_i_*) is a not an upper but a *lower *bound for the coefficient of variation of parameter *p_i_*. Clearly, this lower bound cannot be used to infer identifiability, but it can be used to infer that a model is *not *identifiable.

Both $\bar{v}$ and the significance level for *P*(*χ*^2^|*v*) obviously are adjustable parameters. As discussed in the section entitled *Results and Discussion*, however, the chosen values are not critical. Furthermore, we stress that their values (0.01 and 0.1%, respectively) are conservative in any case.

#### Testing goodness of fit

The purpose of the identifiability tests and variance analyses is to ensure a balance between the number of unknown parameters that need to be estimated on the one hand, and the information content of the data used for their estimation on the other hand. Essentially, we consider model complexity and information content of data to be balanced, if the parameter estimation yields unique values for all estimated parameters, or equivalently, sufficiently small coefficients of variation for all estimated parameters. Besides guaranteeing the uniqueness of the estimated parameters, however, we also need a measure for the quality of the fit of different models to the data. We use a criterion based on Akaike's information criterion [43] for this purpose. The AIC reads

(9) $$AIC=-2\text{ln(}L(g(\hat{p}\text{​})|\text{​}data\text{)}\;\text{+}\;\text{2}n_{p},$$

where $L(g(\hat{p}\text{​})\;|\;\text{​}data\text{)}$ is the likelihood of model $g(\hat{p})$ given the measurement *data*. For brevity $L(g(\hat{p}\text{​})\;|\;\text{​}data\text{)}$ will be denoted as *L*. In case of *χ*^2^-parameter estimation *L *is given by

$$L=\prod_{i=1}^{n_{y}}\prod_{j=1}^{n_{t}}{\left(\frac{1}{2\pi \sigma_{ij}^{2}}\right)}^{\frac{1}{2}}\exp\left(-\sum_{i=1}^{n_{y}}\sum_{j=1}^{n_{t}}\frac{{\left({\tilde{y}}_{i}(t_{j})-y_{i}(t_{j})\right)}^{2}}{2\sigma_{ij}^{2}}\right),$$

and ln(*L*) can be written as

(10) ln(L)=ln(∏i=1ny∏j=1nt(12πσij2)12)−∑i=1ny∑j=1nt(y˜i(tj)−yi(tj))22σij2=C−χ2/2.

For a review see [6,44]. In equation (10) *C *denotes a model independent constant, which can be neglected when comparing AIC values of different models. Combining equations (9) and (10) the AIC criterion can be written as: *AIC *= *χ*^2 ^+ 2*n_p_*. We use a variant of AIC, AICc [45], which is well suited for estimation with small data sets. The results obtained with AICc are compared to other established variants of the AICc criterion, the AICc difference Δ*_k _*and the AICc weights *w_k_*. Δ*_k _*describes the difference between the AICc value of model *M^k ^*and the model with the smallest AICc value. The value of *w_k _*can be interpreted as a probability that model *M^k ^*is the best model within a set of alternative models. A closer description of the criteria can be found in Additional file 1 supplementary text 3. The interested reader is referred to [44] for more details.

#### Model simplification work flow

Having presented its building blocks, the work flow for the model simplification can now be stated. We assume the original model *M*^0 ^is of the form (1). This implies the output variables *y *have been chosen and initial conditions *x*(0) = *x*_0 _are given.

0. Choose the input function *u*(*t*), $t\in [0,t_{n_{t}}]$ Choose an upper bound $\bar{v}$ for the coefficients of variation as introduced in the section *Variance analysis*. Set the iteration counter to *k *= 0.

1. Calculate an estimate ${\hat{p}}^{k}$ for *M^k ^*as described in the section entitled *Parameter estimation*.

2. Calculate the Hessian matrix of *M^k ^*evaluated at the estimated values ${\hat{p}}^{k}$ and rank the parameters of *M^k ^*as described in the section entitled *Identifiability analysis*.

3. For *i *∈ {1,..., *n_p_*} in the order that results from step 2, calculate the coefficient of variation *v*(*p_i_*) as described in the section entitled *Variance analysis*. If $v(p_{i})>\bar{v}$, go to step 4. If $v(p_{i})\leq \bar{v}$ for all *i *= 1,..., *n_p_*, terminate.

4. Simplify those parts of the model that involve the least identifiable parameters. Refer to the resulting model as *M*^*k*+1^, increment *k*, and go to step 1.

As pointed out before [2], the model simplification step (step 4) cannot be carried out automatically, but requires insight into the model. Typical simplifications consists of lumping of reactions or introducing simplified reactions.

Note that the work flow does not guarantee that the smallest eigenvalue of the Hessian matrix increases in every iteration. Since the model structure is changed in step 4, the size and structure of the Hessian matrix changes. Consequently, there is no one-to-one relationship between the eigenvalues of the Hessian before and after a simplification step. In practice, however, a decrease of the smallest eigenvalue from one simplification step to the next rarely occurs. In the example treated here, a decrease occurs in step 5 from *λ*_min _= 6.9 · 10^-7 ^to *λ*_min _= 1.5 · 10^-10^. This decrease is outweighed by an overall increase from *λ*_min _= 1.9 · 10^-15 ^before the first simplification step to *λ*_min _= 1.2 · 10^-6 ^after the last simplification step. Our improved work flow differs from the one proposed in [2] in several aspects. Most importantly, the single step model simplification described in [2] is extended to a multi-step model simplification work flow in which new estimations are carried out after each model simplification step. Secondly, we propose a variance based stopping criterion, which can be interpreted more easily than the eigenvalue bound used in [2]. For both the parameter estimation and the variance analysis step we use multi-start estimations. Finally, the parameter estimation and identifiability analysis are carried out with respect to outputs *y *here. In contrast, trajectories of all state variables *x_i_*(*t*), *i *= 1,..., *n_x _*were assumed to be available in [2]. As pointed out above it is crucial to consider outputs instead of state variables if simulations are supposed to mimic realistic laboratory experiments.

## Results and Discussion

In this section we apply the model simplification work flow to the truncated JAK-STAT model from Figure 1. We refer to this model and the parameter values published by Yamada et al. [36] as *M*^0 ^and ${\hat{p}}^{0}$, respectively. We carry out a sequence of 6 simplification steps. The resulting models and the corresponding estimated parameter values are denoted by *M*^1^,...,*M*^6 ^and ${\hat{p}}^{1},...,{\hat{p}}^{6}$, respectively. All models are described in detail in Additional file 1.

All parameter estimation steps (step 1 of the work flow) are carried out in logarithmic parameter space to efficiently cover a large search area (cf. [31]). When estimating parameters *p^k ^*for model *M^k^*, estimated parameter values ${\hat{p}}^{k-1}$ of *M*^*k*-1 ^serve as reference values. More specifically, starting points *p^k ^*for multi-start parameter estimation are sampled within four magnitudes around these reference values, i.e., in the ranges ${\hat{p}}_{i}^{k-1}\cdot 10^{-2}\leq {\hat{p}}_{i}^{k}\leq {\hat{p}}_{i}^{k-1}\cdot 10^{2}$, *i *= 1,...,*n_p_*. Data points for the estimation are generated once by simulating the reference model *M*^0 ^and recording the output values *y*(*t_j_*), *t*_0 _= 0 min,..., *t*_15 _= 15 min. As proposed in [31] we assume 20% standard deviation for experiments and set *σ_ij _*in Eq. (6) accordingly. For the variance analysis (step 3 of the work flow) we choose the boundaries ${\hat{p}}_{i}\cdot 10^{-\frac{1}{2}}\leq pi\leq {\hat{p}}_{i}\cdot 10^{\frac{1}{2}}$ and a significance level for *P*(*χ*^2^|*v*) of 0.1%. Both choices turned out not to be critical as discussed at the end of the section. Finally, we set $\bar{v}=0.01$. This implies we consider two values for the same parameter to be equal, if these values deviate by a relative error of 1% or less.

In each parameter estimation step and in each variance analysis step 1000 starting points are sampled. The best estimates of each work flow iteration are listed in Additional file 1 table S1.

### Simplification 1: Neglecting the STAT1Phos reassociation to the activated receptor

Values ${\hat{p}}^{0}$ for the parameters of model *M*^0 ^are available from the literature [36] and therefore need not be estimated in the first simplification step. The work flow is therefore started with the identifiability analysis (step 2). This analysis yields a smallest eigenvalue of 1.9 · 10^-15^, which is a strong indication that the model is not identifiable. This result is corroborated in step 3 (variance analysis), where a high coefficient of variation, *v*(kf7) ≥ 0.63, results for the least identifiable parameter, kf7. We can only infer a lower bound on *v*(kf7), since the parameter values estimated in the variance analysis step span the entire range of the starting values. As discussed in the section entitled *Variance analysis*, this indicates that starting values would have to be sampled from a larger parameter space region, if a value for *v *rather than a lower bound was necessary. A lower bound suffices, however, to infer that the variances are too large to consider the model to be identifiable. Consequently, we attempt to simplify those parts of the model that involve the least identifiable parameter kf7 (step 4).

**Figure 3:**
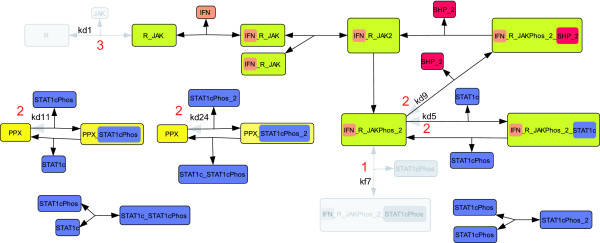
**Visualization of the first three model simplifications**. Removed components are shown in gray. Red numbers indicate the iteration *k *of the work flow in which the reaction has been removed. Unidentifiable parameters are stated next to the respective reaction. In the first iteration the reactions containing kf7, kd7 and the species IFN_R_JAKPhos_2_STAT1cPhos have been removed. In the second iteration the dissociation reactions containing the parameters kd5, kd9, kd11, and kd24 have been removed. In the third iteration the model was simplified by deleting the species R and JAK and the corresponding dissociation and association reactions. A mathematical representation of the models created in each iteration is given in Additional file 1.

The parameter kf7 describes the association of phosphorylated STAT1c to the activated receptor complex (see Figure 3). As discussed in [2] this re-association of previously phosphorylated STAT1c can be neglected, since the phosphorylation of cytoplasmic STAT1c by the activated receptor is the key event rather than the reassociation of the previously phosphorylated STAT1c. Moreover, phosphorylated STAT1cPhos monomers are driven to homodimerize. Therefore, only few such monomers are available to react with the activated receptor. By removing kf7, the backwards reaction with kinetic constant kd7 and the species IFN_R_JAKPhos_2_STAT1cPhos become obsolete and are removed, too. Since the initial value of IFN_R_JAKPhos_2_STAT1cPhos is zero in the reference model, this species can be removed from the model without the need for adjusting the initial values of the remaining state variables. We reduce our model from 17 states and 25 parameters to 16 states and 23 parameters. We refer to the resulting model as *M*^1^.

### Simplification 2: Neglecting the dissociation of high affinity complexes before phosphorylation or dephosphorylation can occur

The unknown model parameters of the simplified model *M*^1 ^are determined by carrying out a multi-start parameter estimation (step 1 of the work flow). The best estimate of the multi-start parameter estimation ${\hat{p}}^{1}$ results in a *χ*^2 ^value of 8.7 · 10^-6 ^and an AICc value of 77 (see Table 1). Both values will be discussed below once a comparison with other models is possible.

**Table 1:** Identifiability ranking and results of multi-start estimation and variance analysis

Model	Top ten parameters of the identifiability ranking	*χ*^2^	AICc	v(p_i_)		
*M*^0^	kf7, kf9, kd11, kd9, kf24, kf13, kd5, kd13, kd7, kf1	none	none	*v*(kf7)	≥ 0.63	>$\bar{v}$
*M*^1^	kd5, kf9, kd11, kf13, kf1, kf25, kd13, kd9, kd1, kd24	8.7 · 10^-6^	77	*v*(kd5)	≥ 0.6	>$\bar{v}$
*M*^2^	kd1, kf25, kf1, kf10, kd3, kf12, kf13, k24, kd13, k11	1.0 · 10^-4^	57	*v*(kd1)	≥ 0.59	>$\bar{v}$
*M*^3^	kf25, kf10, kf12, k24, kd3, kf13, kd13, k11, k9, kf3	9.5 · 10^-5^	49	*v*(kf25)	≥ 0.75	>$\bar{v}$
*M*^4^	k5, kf10, kd3, k9, kf3, kd8, kf4, k11, kf8, kd2	2.7 · 10^-3^	31	*v*(k5)	≥ 0.47	>$\bar{v}$
*M*^5^	k11, kf10, kd2, kf3, kd3, kd8, k9, kf4, kf8, kf2	7.6 · 10^-1^	28	*v*(k11)	≥ 0.18	>$\bar{v}$
*M*^6^	kf10, kd2, kf3, kd3, kd8, k9, kf4, kf8, kf2, k5new	7.6 · 10^-1^	25	*v*(*p_i_*)	≤0.0091	<$\bar{v}$

The first parameter in each ranking is the least identifiable one. No *χ*^2 ^and AICc values are given for *M*^0^, since the model parameters are not estimated but taken from the literature [36].

The identifiability analysis (step 2) and the variance analysis (step 3) result in a smallest eigenvalue of 6.7 · 10^-11 ^and in *v*(kd5) ≥ 0.6 for the least identifiable parameter kd5, respectively. Note that a lower bound results for *v *for the same reason as in the first simplification step. Again this lower bound suffices to infer that the model is not identifiable. Since the identifiability analysis ranks parameter kd5 as the least identifiable parameter, we attempt to simplify the reactions that are affected by kd5.

The parameter kd5 describes the dissociation of unphosphorylated STAT1c from the activated receptor complex. We assume the affinity of STAT1c for the active receptor complex is high [46], or equivalently, phosphorylation of STAT1c on average takes place much faster than the dissociation of unphosphorylated STAT1c. Therefore, the dissociation of unphosphorylated STAT1c is a rare event and the dissociation reaction can be removed from the model.

The same line of argumentation holds for the parameters kd11, kd9, and kd24. kd11 (kd24) describes the dissociation of STAT1cPhos (STAT1cPhos2) from PPX before PPX-induced dephosphorylation occurs. kd9 belongs to the dissociation reaction of SHP2 from the activated receptor that takes place before SHP2 dephosphorylates the activated receptor. For consistency we renamed kf5, kf11, kf9, and kf24 to k5, k11, k9, and k24, respectively. Since no state variables are removed in this simplification the initial conditions are taken from the previous model.

While the resulting model *M*^2 ^has the same number of state variables (16) as the previous model *M*^1^, the number of parameters dropped from 23 in *M*^1 ^to 19 in the new model *M*^2^.

### Simplification 3: Assuming JAK and the receptor are preassociated

The third simplification step starts with the multi-start estimation of the parameters *p*^2 ^of *M*^2 ^(step 1 of the work flow). These parameters can be estimated with a *χ*^2 ^value of 1.0 · 10^-4^. The AICc value decreases from 77 to 57, which implies that the simplifications made so far improve the balance between model detailedness and quality of fit (Table 1).

The identifiability analysis (step 2) indicates, however, that *M*^2 ^with a smallest eigenvalue of 1.2 · 10^-9 ^is not identifiable. This result is confirmed by the variance analysis (step 3), which results in *v*(kd1) ≥ 0.59 for the least identifiable parameter kd1 (Table 1). This value of *v*(kd1) is a lower bound only; see the discussion in the first and second simplification steps. The results of the identifiability analysis and the variance analysis suggest to simplify the reactions that kd1 is involved in.

kd1 is the kinetic constant for the dissociation of JAK from a single receptor. If we remove this reaction, we can further simplify the model by assuming that JAK and the receptor are associated before the signal arrives [47,48] (see Figure 3). The changes amount to assuming that the association of JAK to R is much faster than the dissociation of R_JAK. Under this assumption JAK (JAK(0) = 12 nM) and R (R(0) = 12 nM) initially only exist in complex, therefore the initial condition of R_ JAK has to be changed from 0 to 12 nM.

The resulting model, which we refer to as *M*^3^, comprises 14 state variables and 17 parameters.

### Simplification 4: Omitting PPX mediated STAT1cPhos dephosphorylation

The multi-start parameter estimation for the parameters *p*^3 ^of *M*^3 ^results in *χ*^2 ^= 9.5 · 10^-5 ^(step 1). The AICc value improves from 57 to 49 (cf. Table 1). Step 2 of the work flow ranks kf25 as the least identifiable parameter with a corresponding eigenvalue of 7.0 · 10^-9^, which indicates that kf25 is not identifiable. Step 3 yields a lower bound on the variation, *v*(kf25) ≥ 0.75 for kf25. (cf. Table 1). While providing only a lower bound, this result corroborates that the model needs to be simplified with respect to the reaction kf25 is involved in.

**Figure 4:**
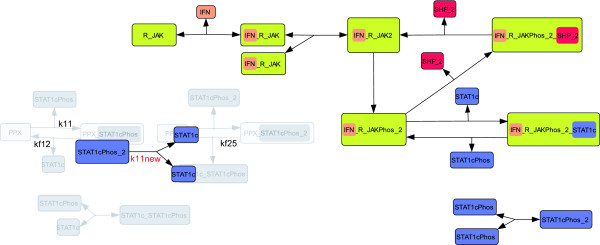
**Simplification of the PPX part leads to model *M*^4^**. The same notation as in Figure 3 is used. Additionally, a red colored parameter name indicates, the introduction of a new parameter. In this simplification step PPX and all corresponding reactions have been removed from the model. In order to still account for STAT1cPhos_2 dissociation and phosphorylation, we added a new dissociation reaction containing the parameter k11new. The mathematical description of model *M*^4 ^is given in Additional file 1 table S6a and S6b.

kf25 describes the rate of the dephosphorylation of STAT1cPhos_2 by PPX (cf. Figure 4). STAT1cPhos can be dephosphorylated by PPX along either of two routes. 1) PPX binds and dephosphorylates the phosphorylated STAT1c monomer or 2) PPX binds the homodimer of phosphorylated STAT1c and subsequently dephosphorylates one of the two STAT1c proteins. In the second case the heterodimer STAT1c_STAT1cPhos is created. Since neither STAT1c heterodimers, nor the phosphatase PPX have been experimentally validated we propose to considerably simplify this part of the model (see Figure 4). We remove PPX and all its complexes from the model and assume a simple first order kinetic for the conversion of the phosphorylated dimer into two unphosphorylated monomers. We stress that removing PPX is a drastic simplification. The fact that the PPX-related parameters kf12 and k24 are ranked third and fourth in the identifiability ranking (cf. Table 1) further suggests that this simplification is reasonable. Since not only PPX (PPX(0) = 50 nM) but also all PPX bound species are removed, the initial conditions of the remaining species do not change.

The resulting model *M*^4 ^is visualized in Figure 4. The number of state variables and parameters drops from 14 to 10 and from 17 to 12, respectively.

### Simplification 5: Combining STAT1c-receptor complex formation, STAT1c phosphorylation, and complex dissociation

The parameter values ${\hat{p}}^{4}$ can be estimated with a *χ*^2 ^value of 2.7 · 10^-3 ^(step 1 of the work flow). The AICc value improved from 49 to 31 (cf. Table 1).

The identifiability analysis ranks k5 as the least identifiable parameter with a corresponding eigenvalue of 6.9 · 10^-7 ^(step 2). The variance analysis results in *v*(*k*5) ≥ 0.47 for k5 (step 3), where *v*(*k*5) is a lower bound for the same reason as in the previous simplification steps. Both the smallest eigenvalue and the large lower bound on *v*(*k*5) indicate that model *M*^4 ^is not identifiable.

**Figure 5:**
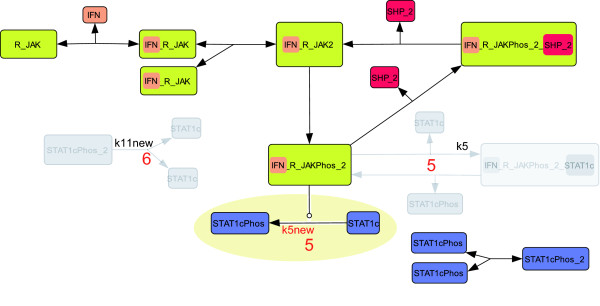
**Visualization of simplifications 5 and 6**. See Figure 3 and 4 for comments on the notation. In iteration 5 the association and dissociation plus phosphorylation of the active receptor IFN_R_JAKPhos_2_STAT1c and STAT1c are replaced by a simplified reaction (highlighted in yellow). In the simplified reaction with the new parameter k5new, phosphorylation of STAT1c by the active receptor is modeled without considering complex formation of both species. In the last iteration the dissociation and dephosphorylation of STAT1cPhos_y2 is removed from the model. A mathematical description of the models created in steps 5 and 6 are given in Additional file 1.

The reactions around k5 are simplified as sketched in Figure 5. Essentially, this simplification combines the formation of the STAT1c-receptor complex, the phosphorylation of STAT1c, and the dissociation into one step. As a consequence, STAT1c phosphorylation is modeled as a second order kinetic in which the activated receptor is not consumed. This implies that STAT1c phosphorylation still depends on the concentration of both the activated receptor complex and STAT1c. In this simplification the species IFN_R_JAKPhos_2_STAT1c is removed. Since initially this species does not exist, its removal has no influence on the remaining initial conditions.

The resulting Model *M*^5 ^has 9 state variables and 11 parameters.

### Simplification 6: Cytoplasmic STAT1cPhos dephosphorylation can be omitted when modeling only the first 15 minutes of signaling

The multi-start parameter estimation for the parameters *p*^5 ^of model *M*^5 ^yields a *χ*^2 ^value of 7.6 · 10^-1 ^(step 1). The AICc value improves from 31 to 28 (cf. Table 1).

The identifiability analysis (step 2) yields a smallest eigenvalue of 1.5 · 10^-10 ^caused by k11, which indicates that model *M*^5 ^is not identifiable. This result is corroborated by a large lower bound on the coefficient of variation of k11, *v*(k11) ≥ 0.18 (step 3). Step 3 does not yield a value but only a lower bound for the coefficient of variation for the same reasons as discussed in the previous simplification steps. k11 is the kinetic constant of the dephosphorylation of STAT1c, a reaction we cannot simplify further. In order to create an identifiable model, we must remove this reaction (see Figure 5), which amounts to ignoring cytoplasmic dephosphorylation in the first 15 minutes of signaling. We stress this does not imply that no cytoplasmic dephosphorylation exists. Note that a similar conclusion was drawn by Yamada et al. [36], who state that dephosphorylation of cytoplasmic STAT by PPX is of minor importance in the pathway. Since no states are removed in this step, the initial conditions are taken from the previous model. While the number of state variables remained unchanged, the number of parameters of *M*^6 ^decreased from 11 to 10.

### Properties of the final model *M*^6^

The multi-start parameter estimation of parameter values *p*^6 ^of model *M*^6 ^results in a *χ*^2 ^value of 7.6 · 10^-1^. Note this is the same value as in the previous simplification step. The AICc value, in contrast, improved from 28 to 25 (cf. Table 1).

Model *M*^6 ^turns out to be identifiable in steps 2 and 3 of the work flow. While this is not clearly apparent from the smallest eigenvalue (1.2 · 10^-6^), the coefficient of variation is now bounded above by $\bar{v}=0.01$ for all parameters. This implies that the multi-start parameter estimation conducted in the variance analysis resulted in parameter values that are within 1% of the respective mean ${\bar{p}}_{i}$ for all parameters *p_i _*of the model.

### Assessing the quality-of-fit with Akaike's information criterion and related criteria

The AICc values improved in all simplification steps. In addition to the AICc values we report AIC values, AICc differences Δ*_k_*, and AIC weights *w_k _*in Table 2. In summary, all criteria agree in the sense that the final model *M*^6 ^is the best model with respect to the quality-of-fit. We note that the improvement of AIC and AICc values is quite low in the last step while the last model has a drastically higher probability of being the "best" model in the sense of its AIC weight *w*_6 _= 78%. We conclude that the resulting simplified model *M*^6 ^is both identifiable, and it balances quality-of-fit and model detailedness.

**Table 2:** Summary of model selection statistics

Model	*n_y_*	*n_p_*	AIC	AICc	Δ*_k_*	*wk*
*M*^1^	16	23	46	77	51	5.3 · 10^-12^
*M*^2^	16	19	38	57	32	9.9 · 10^-8^
*M*^3^	14	17	34	49	23	0.67 · 10^-5^
*M*^4^	10	12	24	31	5.4	0.52 · 10^-1^
*M*^5^	9	11	23	28	3.0	0.17
*M*^6^	9	10	21	25	0	0.78

### Choice of parameter boundaries and the significance level in the variance analysis

The starting values for the variance analysis must be chosen from a sufficiently large parameter space neighborhood of the nominal values $\hat{p}$. If the estimates obtained in the variance analysis converge to values that cover the entire sampled neighborhood, the coefficient of variation may be limited by the choice of the neighborhood. Since enlarging the sampling neighborhood can only result in equal or larger coefficients of variation, the *v *values obtained with a too small neighborhood are lower bounds on the actual coefficients of variation.

In all but the last simplification step the estimates obtained in the variance analyses cover the entire sampling neighborhood (data not shown). The resulting lower bounds on *v *are sufficiently large (cf. Table 1), however, to infer that the respective models are not identifiable. In the final simplification step, in contrast, the sampling neighborhood is large enough, since the estimates obtained in the variance analysis are tightly packed within 1% error ($\bar{v}=0.01$, cf. Table 1) around the nominal value. The choice of the sampling neighborhood is therefore not critical in any simplification step.

We claim the chosen value of 0.1% for the significance level is not critical, either, since the work flow terminates after the same number of simplification steps for a large range of values, which can be seen as follows. The values of *v *do not change if the significance level is increased in the sixth simplification step, since all converged parameter estimates for ${\hat{p}}^{6}$ (970 out of 1000) are already accepted for the chosen value 0.1%. Smaller, i.e. stricter, significance levels well below 10^-10^, on the other hand, do not change the coefficients of variation, either (data not shown). In the first through fifth simplification step, the coefficients of variation increase for larger, i.e. less strict, significance levels than 0.1%. Since the coefficients of variation are large enough to necessitate another iteration through the work flow already, increasing the significance level does not have any effect. On the other hand, decreasing the significance level to value as low as 10^-9 ^does not affect the *v*-values for *M*^1 ^through *M*^5 ^(data not shown).

## Conclusions

We presented a work flow for model simplification and demonstrated its use by simplifying an overly detailed model for JAK-STAT signal transduction. Here, we used simulated data from a model taken from the literature. However, the method is intended to be used with real data. The work flow can be used to ensure model quality with respect to two important aspects: 1) Given an unidentifiable model, the work flow results in a model that is locally identifiable with small coefficients of variation for all parameters and 2) the balance of goodness of fit and the amount of model detail - as quantified by AIC based criteria - is better than for the original model and intermediate models that arise in the work flow. The first point is addressed by applying both local identifiability analysis and local multi-start parameter estimation with subsequent analysis of the variance of acceptable estimates. The second point is taken care of by calculating the information criteria, based on AIC, that provide a measure for the information loss that occurs when reducing model complexity.

The proposed work flow does not automate model simplifications, but the identifiability ranking of parameters only provides hints on which parts of the model are too detailed. Nevertheless, the simplifications found for the JAK-STAT example lend themselves to biologically sound interpretations. For example, dissociation reactions are neglected for high affinity complexes in the simplified model (second simplification), JAK and the receptor (R) are assumed to be preassociated (third simplification), and cytoplasmic dephosphorylation turns out not to be relevant for the considered outputs (fourth and sixth simplification).

Several other authors suggest to fix unidentifiable parameters and to estimate the remaining ones [3,31-34]. In contrast, the model structure is simplified in the present paper until all parameters are identifiable, i.e. all parameters can be estimated with small error bars. Note that changes in the model structure imply structural changes of the Hessian matrix. Due to these changes together with the local character of the identifiability method, *λ*_min _may decrease after one simplification step. In practice, however, this is a rare event, which is outweighed by a prominent increasing trend of *λ*_min_.

We stress that the presented case study involves generating output data points *y_i_*(*t_j_*) by simulations carried out with the unidentifiable reference model *M*^0^. In this sense, both parameter fixing, and the approach as used in the case study involve unidentifiable models. However, when real experimental data is used, our approach is free of this defect. While more involved than parameter fixing, the proposed approach results in models with a complexity that is consistent with the experimentally accessible data.

## Authors' contributions

TQ and MM developed the presented work flow. TQ implemented the method and accomplished the model simplification of the JAK-STAT pathway. AD and FS designed the hypothetical experiments and made critical suggestions to model simplifications. All authors drafted, read and approved the final manuscript.

## Supplementary Material

Additional file 1 **The supplementary pdf file accompanying this article contains the supplementary tables S1-S8b and supplementary texts 1-3**. Click here for file
