# Supplementary material for: A simple work flow for biologically inspired model reduction - application to early JAK-STAT signaling
Source: BMC Syst Biol. 2011 Feb 21;5:30. doi: 10.1186/1752-0509-5-30 (PMC3050741; doi:10.1186/1752-0509-5-30)
Supplement: Additional file 1 — The supplementary pdf file accompanying this article contains the supplementary tables S1-S8b and supplementary texts 1-3. [file 1752-0509-5-30-S1.PDF]

Supplementary material:
A simple work flow for biologically inspired model reduction – application to early JAK-STAT signaling

Tom Quaiser, Anna Dittrich, Fred Schaper, Martin Mönnigmann*

Email: Martin Mönnigmann*- martin.moennigmann@rub.de;

*Corresponding author

Table S1 - Reference parameters from literature $\hat{p}^0$ and estimates $\hat{p}^1 - \hat{p}^6$

	Parameter values (s^{-1} for dissociation and $nM^{-1} \cdot s^{-1}$ for dimerization constants)
$\hat{p}^0$	kf1 = 0.1, kd1 = $0.5 \cdot 10^{-1}$, kf2 = $0.2 \cdot 10^{-1}$, kd2 = $0.2 \cdot 10^{-1}$, kf3 = $0.4 \cdot 10^{-1}$, kd3 = 0.2, kf4 = $0.5 \cdot 10^{-2}$, kf5 = $0.8 \cdot 10^{-2}$, kd5 = 0.8,kf6 = 0.4, kf7 = $0.5 \cdot 10^{-2}$, kd7 = 0.5, kf8 = $0.2 \cdot 10^{-1}$, kd8 = 0.1, kf9 = $0.1 \cdot 10^{-2}$, kd9 = 0.2, kf10 = $0.3 \cdot 10^{-2}$, kf11 = $0.1 \cdot 10^{-2}$, kd11 = 0.2, kf12 = $0.3 \cdot 10^{-2}$, kf13 = $2. \cdot 10^{-7}$, kd13 = 0.2, kf24 = $0.1 \cdot 10^{-2}$, kd24 = 0.2, kf25 = $0.3 \cdot 10^{-2}$
$\hat{p}^1$	kf1 = $0.76 \cdot 10^{-1}$, kd1 = $0.26 \cdot 10^{-1}$, kf2 = $0.25 \cdot 10^{-1}$, kd2 = $0.88 \cdot 10^{-1}$, kf3 = $0.29 \cdot 10^{-1}$, kd3 = $0.20 \cdot 10^{-1}$, kf4 = $0.45 \cdot 10^{-2}$, kf5 = $0.15 \cdot 10^{-1}$, kd5 = 0.93, kf6 = 0.48, kf8 = $0.15 \cdot 10^{-1}$, kd8 = $0.96 \cdot 10^{-1}$, kf9 = $0.16 \cdot 10^{-1}$, kd9 = 0.39, kf10 = $0.83 \cdot 10^{-3}$, kf11 = $0.52 \cdot 10^{-4}$, kd11 = $0.23 \cdot 10^{-2}$, kf12 = $0.47 \cdot 10^{-2}$, kf13 = $0.2 \cdot 10^{-8}$, kd13 = $0.90 \cdot 10^{-2}$, kf24 = $0.26 \cdot 10^{-3}$, kd24 = $0.17 \cdot 10^{-1}$, kf25 = $0.76 \cdot 10^{-3}$
$\hat{p}^2$	kf1 = 0.59, kd1 = $0.12 \cdot 10^{-2}$, kf2 = $0.17 \cdot 10^{-1}$, kd2 = $0.15 \cdot 10^{-1}$, kf3 = $0.64 \cdot 10^{-1}$, kd3 = 0.77, kf4 = $0.61 \cdot 10^{-2}$, k5 = $0.12 \cdot 10^{-2}$, kf6 = 0.47, kf8 = $0.11 \cdot 10^{-1}$, kd8 = $0.56 \cdot 10^{-1}$, k9 = $0.67 \cdot 10^{-5}$, kf10 = $0.14 \cdot 10^{-1}$, k11 = $0.68 \cdot 10^{-4}$, kf12 = $0.19 \cdot 10^{-1}$, kf13 = $9.9 \cdot 10^{-7}$, kd13 = $0.89 \cdot 10^{-2}$, k24 = $0.50 \cdot 10^{-5}$, kf25 = $0.30 \cdot 10^{-4}$
$\hat{p}^3$	kf2 = $0.17 \cdot 10^{-1}$, kd2 = $0.15 \cdot 10^{-1}$, kf3 = $0.41 \cdot 10^{-1}$, kd3 = 0.52, kf4 = $0.62 \cdot 10^{-2}$, k5 = $0.12 \cdot 10^{-2}$, kf6 = 0.47, kf8 = $0.11 \cdot 10^{-1}$, kd8 = $0.56 \cdot 10^{-1}$, k9 = $0.68 \cdot 10^{-5}$, kf10 = $0.13 \cdot 10^{-1}$, k11 = $0.68 \cdot 10^{-4}$, kf12 = $0.18 \cdot 10^{-1}$, kf13 = $0.1 \cdot 10^{-8}$, kd13 = $0.82 \cdot 10^{-2}$, k24 = $0.48 \cdot 10^{-5}$, kf25 = $0.30 \cdot 10^{-4}$
$\hat{p}^4$	kf2 = $0.17 \cdot 10^{-1}$, kd2 = $0.14 \cdot 10^{-1}$, kf3 = $0.78 \cdot 10^{-1}$, kd3 = 1.3, kf4 = $0.73 \cdot 10^{-2}$, k5 = $0.15 \cdot 10^{-1}$, kf6 = 0.33, kf8 = $0.14 \cdot 10^{-1}$, kd8 = $0.82 \cdot 10^{-1}$, k9 = $0.84 \cdot 10^{-4}$, kf10 = $0.73 \cdot 10^{-2}$, k11 = $0.41 \cdot 10^{-3}$
$\hat{p}^5$	kf2 = $0.19 \cdot 10^{-2}$, kd2 = $0.17 \cdot 10^{-1}$, kf3 = 0.34, kd3 = $0.21 \cdot 10^{-1}$, kf4 = $0.23 \cdot 10^{-1}$, kf8 = $0.14 \cdot 10^{-1}$, kd8 = $0.91 \cdot 10^{-1}$, k9 = $0.12 \cdot 10^{-4}$, kf10 = 0.30, k11 = $1.0 \cdot 10^{-7}$, k5new = $0.35 \cdot 10^{-3}$
$\hat{p}^6$	kf2 = $0.19 \cdot 10{-}2$, kd2 = $0.17 \cdot 10{-}1$, kf3 = 0.34, kd3 = $0.21 \cdot 10{-}1$, kf4 = $0.23 \cdot 10{-}1$, kf8 = $0.14 \cdot 10{-}1$, kd8 = $0.91 \cdot 10{-}1$, k9 = $0.12 \cdot 10{-}4$, kf10 = 0.30, k5new = $0.35 \cdot 10{-}3$

Table S2a - Variables and initial values of Model M^0

Variables	Description	Initial values (nM)
x_1	R	12
x_2	JAK	12
x_3	R_ JAK	0
$x_4 \rightarrow u$	IFN	1
x_5	IFN_ R_ JAK	0
x_6	IFN_ R_ JAK2	0
x_7	IFN_ R_ JAKPhos_ 2	0
x_8	IFN_ R_ JAKPhos_ 2_ STAT1cPhos	0
x_9	STAT1cPhos	0
x_{10}	STAT1cPhos_ 2	0
x_{11}	STAT1c	1000
x_{12}	IFN_ R_ JAKPhos_ 2_ STAT1c	0
x_{13}	PPX_ STAT1cPhos	0
x_{14}	STAT1c_ STAT1cPhos	0
x_{15}	PPX_ STAT1cPhos_ 2	0
x_{16}	IFN_ R_ JAKPhos_ 2_ SHP_ 2	0
x_{17}	PPX	50
x_{18}	SHP_ 2	100

Table S2b - Rate equations and ODE system of M^0

Rate equations	Differential equations
$v_1 = kf1 \cdot x_1 \cdot x_2 - kd1 \cdot x_3$	$\dot{x}_1 = -v_1$
$v_2 = kf2 \cdot x_3 \cdot IFN - kd2 \cdot x_5$	$\dot{x}_2 = -v_1$
$v_3 = kf3 \cdot x_5^2 - kd3 \cdot x_6$	$\dot{x}_3 = +v_1 - v_2$
$v_4 = kf4 \cdot x_6$	$\dot{x}_5 = +v_2 - 2 \cdot v_3$
$v_5 = kf5 \cdot x_7 \cdot x_{11} - kd5 \cdot x_{12}$	$\dot{x}_6 = +v_3 - v_4 + v_{10}$
$v_6 = kf6 \cdot x_{12}$	$\dot{x}_7 = +v_4 - v_5 + v_6 - v_7 - v_9$
$v_7 = kf7 \cdot x_7 \cdot x_9 - kd7 \cdot x_8$	$\dot{x}_8 = +v_7$
$v_8 = kf8 \cdot x_9^2 - kd8 \cdot x_{10}$	$\dot{x}_9 = +v_6 - v_7 - 2 \cdot v_8 - v_{11} - v_{13}$
$v_9 = kf9 \cdot x_7 \cdot x_{18} - kd9 \cdot x_{16}$	$\dot{x}_{10} = +v_8 - v_{14}$
$v_{10} = kf10 \cdot x_{16}$	$\dot{x}_{11} = -v_5 + v_{12} - v_{13}$
$v_{11} = kf11 \cdot x_9 \cdot x_{17} - kd11 \cdot x_{13}$	$\dot{x}_{12} = +v_5 - v_6$
$v_{12} = kf12 \cdot x_{13}$	$\dot{x}_{13} = +v_{11} - v_{12}$
$v_{13} = kf13 \cdot x_9 \cdot x_{11} - kd13 \cdot x_{14}$	$\dot{x}_{14} = +v_{13} + v_{15}$
$v_{14} = kf24 \cdot x_{17} \cdot x_{10} - kd24 \cdot x_{15}$	$\dot{x}_{15} = +v_{14} - v_{15}$
$v_{15} = kf25 \cdot x_{15}$	$\dot{x}_{16} = +v_9 - v_{10}$
	$\dot{x}_{17} = -v_{11} + v_{12} - v_{14} + v_{15}$
	$\dot{x}_{18} = -v_9 + v_{10}$

Table S3a - Variables and initial values of Model M^1

Variables	Description	Initial values (nM)
x_1	R	12
x_2	JAK	12
x_3	R_ JAK	0
$x_4 \rightarrow u$	IFN	1
x_5	IFN_ R_ JAK	0
x_6	IFN_ R_ JAK2	0
x_7	IFN_ R_ JAKPhos_ 2	0
x_8	STAT1cPhos	0
x_9	STAT1cPhos_ 2	0
x_{10}	STAT1c	1000
x_{11}	IFN_ R_ JAKPhos_ 2_ STAT1c	0
x_{12}	PPX_ STAT1cPhos	0
x_{13}	STAT1c_ STAT1cPhos	0
x_{14}	PPX_ STAT1cPhos_ 2	0
x_{15}	IFN_ R_ JAKPhos_ 2_ SHP_ 2	0
x_{16}	PPX	50
x_{17}	SHP_ 2	100

Table S3b - Rate equations and ODE system of M^1

Rate equations	Differential equations
$v_1 = kf1 \cdot x_1 \cdot x_2 - kd1 \cdot x_3$	$\dot{x}_1 = -v_1$
$v_2 = kf2 \cdot x_3 \cdot IFN - kd2 \cdot x_5$	$\dot{x}_2 = -v_1$
$v_3 = kf3 \cdot x_5^2 - kd3 \cdot x_6$	$\dot{x}_3 = +v_1 - v_2$
$v_4 = kf4 \cdot x_6$	$\dot{x}_5 = +v_2 - 2.0 \cdot v_3$
$v_5 = kf5 \cdot x_7 \cdot x_{10} - kd5 \cdot x_{11}$	$\dot{x}_6 = +v_3 - v_4 + v_9$
$v_6 = kf6 \cdot x_{11}$	$\dot{x}_7 = +v_4 - v_5 + v_6 - v_8$
$v_7 = kf8 \cdot x_8^2 - kd8 \cdot x_9$	$\dot{x}_8 = +v_6 - 2.0 \cdot v_7 - v_{10} - v_{12}$
$v_8 = kf9 \cdot x_7 \cdot x_{17} - kd9 \cdot x_{15}$	$\dot{x}_9 = +v_7 - v_{13}$
$v_9 = kf10 \cdot x_{15}$	$\dot{x}_{10} = -v_5 + v_{11} - v_{12}$
$v_{10} = kf11 \cdot x_8 \cdot x_{16} - kd11 \cdot x_{12}$	$\dot{x}_{11} = +v_5 - v_6$
$v_{11} = kf12 \cdot x_{12}$	$\dot{x}_{12} = +v_{10} - v_{11}$
$v_{12} = kf13 \cdot x_8 \cdot x_{10} - kd13 \cdot x_{13}$	$\dot{x}_{13} = +v_{12} + v_{14}$
$v_{13} = kf24 \cdot x_{16} \cdot x_9 - kd24 \cdot x_{14}$	$\dot{x}_{14} = +v_{13} - v_{14}$
$v_{14} = kf25 \cdot x_{14}$	$\dot{x}_{15} = +v_8 - v_9$
	$\dot{x}_{16} = -v_{10} + v_{11} - v_{13} + v_{14}$
	$\dot{x}_{17} = -v_8 + v_9$

Table S4a - Variables and initial values of Model M^2

Variables	Description	Initial values (nM)
x_1	R	12
x_2	JAK	12
x_3	R_ JAK	0
$x_4 \rightarrow u$	IFN	1
x_5	IFN_ R_ JAK	0
x_6	IFN_ R_ JAK2	0
x_7	IFN_ R_ JAKPhos_ 2	0
x_8	STAT1cPhos	0
x_9	STAT1cPhos_ 2	0
x_{10}	STAT1c	1000
x_{11}	IFN_ R_ JAKPhos_ 2_ STAT1c	0
x_{12}	PPX_ STAT1cPhos	0
x_{13}	STAT1c_ STAT1cPhos	0
x_{14}	PPX_ STAT1cPhos_ 2	0
x_{15}	IFN_ R_ JAKPhos_ 2_ SHP_ 2	0
x_{16}	PPX	50
x_{17}	SHP_ 2	100

Table S4b - Rate equations and ODE system of M^2

Rate equations	Differential equations
$v_1 = kf1 \cdot x_1 \cdot x_2 - kd1 \cdot x_3$	$\dot{x}_1 = -v_1$
$v_2 = kf2 \cdot x_3 \cdot IFN - kd2 \cdot x_5$	$\dot{x}_2 = -v_1$
$v_3 = kf3 \cdot x_5^2 - kd3 \cdot x_6$	$\dot{x}_3 = +v_1 - v_2$
$v_4 = kf4 \cdot x_6$	$\dot{x}_5 = +v_2 - 2.0 \cdot v_3$
$v_5 = k5 \cdot x_7 \cdot x_{10}$	$\dot{x}_6 = +v_3 - v_4 + v_9$
$v_6 = kf6 \cdot x_{11}$	$\dot{x}_7 = +v_4 - v_5 + v_6 - v_8$
$v_7 = kf8 \cdot x_8^2 - kd8 \cdot x_9$	$\dot{x}_8 = +v_6 - 2.0 \cdot v_7 - v_{10} - v_{12}$
$v_8 = k9 \cdot x_7 \cdot x_{17}$	$\dot{x}_9 = +v_7 - v_{13}$
$v_9 = kf10 \cdot x_{15}$	$\dot{x}_{10} = -v_5 + v_{11} - v_{12}$
$v_{10} = k11 \cdot x_8 \cdot x_{16}$	$\dot{x}_{11} = +v_5 - v_6$
$v_{11} = kf12 \cdot x_{12}$	$\dot{x}_{12} = +v_{10} - v_{11}$
$v_{12} = kf13 \cdot x_8 \cdot x_{10} - kd13 \cdot x_{13}$	$\dot{x}_{13} = +v_{12} + v_{14}$
$v_{13} = k24 \cdot x_{16} \cdot x_9$	$\dot{x}_{14} = +v_{13} - v_{14}$
$v_{14} = kf25 \cdot x_{14}$	$\dot{x}_{15} = +v_8 - v_9$
	$\dot{x}_{16} = -v_{10} + v_{11} - v_{13} + v_{14}$
	$\dot{x}_{17} = -v_8 + v_9$

Table S5a - Variables and initial values of Model M^3

Variables	Description	Initial values (nM)
x_1	R_ JAK	12
$x_2 \rightarrow u$	IFN	1
x_3	IFN_ R_ JAK	0
x_4	IFN_ R_ JAK2	0
x_5	IFN_ R_ JAKPhos_ 2	0
x_6	STAT1cPhos	0
x_7	STAT1cPhos_ 2	0
x_8	STAT1c	1000
x_9	IFN_ R_ JAKPhos_ 2_ STAT1c	0
x_{10}	PPX_ STAT1cPhos	0
x_{11}	STAT1c_ STAT1cPhos	0
x_{12}	PPX_ STAT1cPhos_ 2	0
x_{13}	IFN_ R_ JAKPhos_ 2_ SHP_ 2	0
x_{14}	PPX	50
x_{15}	SHP_ 2	100

Table S5b - Rate equations and ODE system of M^3

Rate equations	Differential equations
$v_1 = kf2 \cdot x_1 \cdot IFN - kd2 \cdot x_3$	$\dot{x}_1 = -v_1$
$v_2 = kf3 \cdot x_3^2 - kd3 \cdot x_4$	$\dot{x}_3 = +v_1 - 2.0 \cdot v_2$
$v_3 = kf4 \cdot x_4$	$\dot{x}_4 = +v_2 - v_3 + v_8$
$v_4 = k5 \cdot x_5 \cdot x_8$	$\dot{x}_5 = +v_3 - v_4 + v_5 - v_7$
$v_5 = kf6 \cdot x_9$	$\dot{x}_6 = +v_5 - 2.0 \cdot v_6 - v_9 - v_{11}$
$v_6 = kf8 \cdot x_6^2 - kd8 \cdot x_7$	$\dot{x}_7 = +v_6 - v_{12}$
$v_7 = k9 \cdot x_5 \cdot x_{15}$	$\dot{x}_8 = -v_4 + v_{10} - v_{11}$
$v_8 = kf10 \cdot x_{13}$	$\dot{x}_9 = +v_4 - v_5$
$v_9 = k11 \cdot x_6 \cdot x_{14}$	$\dot{x}_{10} = +v_9 - v_{10}$
$v_{10} = kf12 \cdot x_{10}$	$\dot{x}_{11} = +v_{11} + v_{13}$
$v_{11} = kf13 \cdot x_6 \cdot x_8 - kd13 \cdot x_{11}$	$\dot{x}_{12} = +v_{12} - v_{13}$
$v_{12} = k24 \cdot x_{14} \cdot x_7$	$\dot{x}_{13} = +v_7 - v_8$
$v_{13} = kf25 \cdot x_{12}$	$\dot{x}_{14} = -v_9 + v_{10} - v_{12} + v_{13}$
	$\dot{x}_{15} = -v_7 + v_8$

Table S6a - Variables and initial values of Model M^4

Variables	Description	Initial values (nM)
x_1	R_ JAK	12
$x_2 \rightarrow u$	IFN	1
x_3	IFN_ R_ JAK	0
x_4	IFN_ R_ JAK2	0
x_5	IFN_ R_ JAKPhos_ 2	0
x_6	STAT1cPhos	0
x_7	STAT1cPhos_ 2	0
x_8	STAT1c	1000
x_9	IFN_ R_ JAKPhos_ 2_ STAT1c	0
x_{10}	IFN_ R_ JAKPhos_ 2_ SHP_ 2	0
x_{11}	SHP_ 2	100

Table S6b - Rate equations and ODE system of M^4

Rate equations	Differential equations
$v_1 = kf2 \cdot x_1 \cdot IFN - kd2 \cdot x_3$	$\dot{x}_1 = -v_1$
$v_2 = kf3 \cdot x_3^2 - kd3 \cdot x_4$	$\dot{x}_3 = +v_1 - 2 \cdot v_2$
$v_3 = kf4 \cdot x_4$	$\dot{x}_4 = +v_2 - v_3 + v_8$
$v_4 = k5 \cdot x_5 \cdot x_8$	$\dot{x}_5 = +v_3 - v_4 + v_5 - v_7$
$v_5 = kf6 \cdot x_9$	$\dot{x}_6 = +v_5 - 2 \cdot v_6$
$v_6 = kf8 \cdot x_6^2 - kd8 \cdot x_7$	$\dot{x}_7 = +v_6 - v_9$
$v_7 = k9 \cdot x_5 \cdot x_{11}$	$\dot{x}_8 = -v_4 + 2 \cdot v_9$
$v_8 = kf10 \cdot x_{10}$	$\dot{x}_9 = +v_4 - v_5$
$v_9 = x_7 \cdot k11new$	$\dot{x}_{10} = +v_7 - v_8$
	$\dot{x}_{11} = -v_7 + v_8$

Table S7a - Variables and initial values of Model M^5

Variables	Description	Initial values (nM)
x_1	R_ JAK	12
$x_2 \rightarrow u$	IFN	1
x_3	IFN_ R_ JAK	0
x_4	IFN_ R_ JAK2	0
x_5	IFN_ R_ JAKPhos_ 2	0
x_6	STAT1cPhos	0
x_7	STAT1cPhos_ 2	0
x_8	STAT1c	1000
x_9	IFN_ R_ JAKPhos_ 2_ SHP_ 2	0
x_{10}	SHP_ 2	100

Table S7b - Rate equations and ODE system of M^5

Rate equations	Differential equations
$v_1 = kf2 \cdot x_1 \cdot IFN - kd2 \cdot x_3$	$\dot{x}_1 = -v_1$
$v_2 = kf3 \cdot x_3^2 - kd3 \cdot x_4$	$\dot{x}_3 = +v_1 - 2 \cdot v_2$
$v_3 = kf4 \cdot x_4$	$\dot{x}_4 = +v_2 - v_3 + v_6$
$v_4 = kf8 \cdot x_6^2 - kd8 \cdot x_7$	$\dot{x}_5 = +v_3 - v_5$
$v_5 = k9 \cdot x_5 \cdot x_{10}$	$\dot{x}_6 = -2 \cdot v_4 + v_8$
$v_6 = kf10 \cdot x_9$	$\dot{x}_7 = +v_4 - v_7$
$v_7 = x_7 \cdot k11new$	$\dot{x}_8 = +2.0 \cdot v_7 - v_8$
$v_8 = x_8 \cdot x_5 \cdot k5new$	$\dot{x}_9 = +v_5 - v_6$
	$\dot{x}_{10} = -v_5 + v_6$

Table S8a - Variables and initial values of Model M^6

Variables	Description	Initial values (nM)
x_1	R_ JAK	12
$x_2 \rightarrow u$	IFN	1
x_3	IFN_ R_ JAK	0
x_4	IFN_ R_ JAK2	0
x_5	IFN_ R_ JAKPhos_ 2	0
x_6	STAT1cPhos	0
x_7	STAT1cPhos_ 2	0
x_8	STAT1c	1000
x_9	IFN_ R_ JAKPhos_ 2_ SHP_ 2	0
x_{10}	SHP_ 2	100

Table S8b - Rate equations and ODE system of M^6

Rate equations	Differential equations
$v_1 = kf2 \cdot x_1 \cdot IFN - kd2 \cdot x_3$	$\dot{x}_1 = -v_1$
$v_2 = kf3 \cdot x_3^2 - kd3 \cdot x_4$	$\dot{x}_3 = +v_1 - 2 \cdot v_2$
$v_3 = kf4 \cdot x_4$	$\dot{x}_4 = +v_2 - v_3 + v_6$
$v_4 = kf8 \cdot x_6^2 - kd8 \cdot x_7$	$\dot{x}_5 = +v_3 - v_5$
$v_5 = k9 \cdot x_5 \cdot x_{10}$	$\dot{x}_6 = -2 \cdot v_4 + v_7$
$v_6 = kf10 \cdot x_9$	$\dot{x}_7 = +v_4$
$v_7 = x_8 \cdot x_5 \cdot k5new$	$\dot{x}_8 = -v_7$
	$\dot{x}_9 = +v_5 - v_6$
	$\dot{x}_{10} = -v_5 + v_6$

Supplementary text 1: Simultaneous estimation of unknown parameters and unknown initial conditions
The system class treated in the paper reads

$$\begin{aligned} \dot{x}(t) &= f(x(t), p, u(t)), \quad x(0) = x_0 \\ y(t) &= h(x(t), p, u(t)), \end{aligned} \tag{1}$$

where $x \in \mathbb{R}^{n_x}$, $p \in \mathbb{R}^{n_p}$, $u \in \mathbb{R}^{n_u}$ and $y \in \mathbb{R}^{n_y}$. In particular the initial conditions x_0 were assumed to be known in (1). Here we sketch how the system class can be extended to estimate the initial conditions along with the unknown parameters p. For simplicity we assume that all initial conditions are unknown and must

be estimated. It is, however, straight forward to extend the following procedure to the case, where some but not all initial conditions must be estimated.
Assume $x_0 \in \mathbb{R}^{n_x}$ in equation (1) are unknown and must be estimated. Define $\tilde{x}(t) = x(t) - x_0$ and note that

$$f(x(t), p, u(t)) = f(\tilde{x}(t) + x_0, p, u(t)). \tag{2}$$

Furthermore $\dot{\tilde{x}}(t) = \dot{x}(t)$, and $x(0) = x_0$ is equivalent to $\tilde{x}(0) = 0$. Let $\tilde{p} = (p^t, x_0^t)^t$ and define $\tilde{f} : \mathbb{R}^{n_x} \times \mathbb{R}^{n_p + n_x} \times \mathbb{R}^{n_u} \rightarrow \mathbb{R}^{n_x}$ by

$$\tilde{f}(\tilde{x}(t), \tilde{p}, u(t)) = f(\tilde{x}(t) + x_0, p, u(t)). \tag{3}$$

Then a function $t \rightarrow \tilde{x}(t)$ solves

$$\dot{\tilde{x}}(t) = \tilde{f}(\tilde{x}, \tilde{p}, u(t)), \quad \tilde{x}(0) = 0, \tag{4}$$

if and only if $x(t) = \tilde{x}(t) + x_0$ solves the ODE in equation (1). By the corresponding substitutions the output equations in (1) can be replaced by

$$y(t) = \tilde{h}(\tilde{x}(t), \tilde{p}, u(t)). \tag{5}$$

The new parameters $\tilde{p}$ in (4) and (5), however, comprise the unknown initial conditions. By applying the same methodology for parameter estimation to $\tilde{p}$ as the one applied to p in the paper, both unknown parameters and unknown initial conditions can be estimated simultaneously.

Supplementary text 2: Latin hypercube sampling

LHS is a method to create samples that efficiently cover the sampling space. Since we are interested in sampling the parameter space, the hypercube we are considering has n_p dimensions. For each parameter p_i an interval of feasible values $[p_i^{\text{low}}, p_i^{\text{high}}]$ has to be defined. We divide each interval into n_{lhs} non-overlapping subintervals, where n_{lhs} corresponds to the number of desired samples. For all parameters we now draw a value from each interval with respect to the probability density in the interval. In our case we use a uniform distribution for this step. For each parameter p_i we store the n_{lhs} values in a bin b_i. To create one sample for p, we draw one element of each bin and assign the value to the corresponding p_i. Previously selected elements are not returned to a bin.

Supplementary text 3: AIC based criteria

Below you can find the three AIC based criteria used here, namely AICc, AICc difference (Δ_k) and AICc weights (w_k):

$$AICc = AIC + \frac{2n_p(n_p+1)}{n_y n_t - n_p - 1}$$
$$\Delta_k = AICc_k - AICc_{\min}$$
$$w_k = \frac{\exp(-1/2\Delta_k)}{\sum_{j=1}^{m} \exp(1/2\Delta_j)}.$$

$AICc_{\min}$ and m correspond to the smallest AICc value of all models and the number of models, respectively. AICc has been introduced in [1] and is recommended, when the ratio of the number of samples ($n_y n_t$) to the number of parameters (n_p) is below 40 [2], as is the case here.

References

1. Hurvich C, Tsai C: **Regression and time series model selection in small samples**. Biometrika 1989, **76**(2):297.
2. Burnham K, Anderson D: Model selection and multimodel inference: a practical information-theoretic approach. Springer, New York 2002.
